# Nano-Selenium Alleviates Cd-Induced Chronic Colitis through Intestinal Flora

**DOI:** 10.3390/nu16091330

**Published:** 2024-04-28

**Authors:** Chengdong Zhou, Shengliang Guo, Pin Gong, Qian Ba, Wenbo Yao

**Affiliations:** 1 School of Food Science and Engineering, Shaanxi University of Science and Technology, Xi’an 710021, China; chengdongzhou1004@163.com (C.Z.); guo769698@163.com (S.G.); gongpin@sust.edu.cn (P.G.); 2 Laboratory Center, Shanghai Municipal Hospital of Traditional Chinese Medicine, Shanghai University of Traditional Chinese Medicine, 274 Zhijiang Middle Road, Shanghai 200071, China; 3 Department of Pediatrics, Shanghai Municipal Hospital of Traditional Chinese Medicine, Shanghai University of Traditional Chinese Medicine, 274 Zhijiang Middle Road, Shanghai 200071, China

**Keywords:** cadmium, nano-selenium, intestinal microorganisms, intestinal barrier, inflammatory

## Abstract

Background: Cadmium (Cd) is an environmental contaminant that poses risks to human and animal health. Selenium (Se), a beneficial element, alleviates the detrimental consequences of colitis and Cd toxicity. Se is found in food products as both inorganic Se (sodium selenite) and organic Se (typically Se-enriched yeast). Nano-selenium (nano-Se; a novel form of Se produced through the bioreduction of Se species) has recently garnered considerable interest, although its effects against Cd-induced enterotoxicity are poorly understood. The aim of this study was to investigate the impact of nano-selenium on mitigating cadmium toxicity and safeguarding the integrity of the intestinal barrier. Methods: For a total of two cycles, we subjected 6-week-old C57 mice to chronic colitis by exposing them to Cd and nano-selenium for two weeks, followed by DSS water for one week. Results: The application of nano-selenium mitigated the intensity of colitis and alleviated inflammation in the colon. Nano-selenium enhanced the diversity of the intestinal flora, elevated the concentration of short-chain fatty acids (SCFAs) in feces, and improved the integrity of the intestinal barrier. Conclusions: In summary, nano-Se may reduce intestinal inflammation by regulating the growth of intestinal microorganisms and protecting the intestinal barrier.

## 1. Introduction

Cadmium (Cd) contamination represents one of the most severe forms of heavy metal pollution in China. The detrimental effects of chronic dietary Cd exposure on the human body are a prominent concern in the realm of food safety. Consequently, Cd is classified as a Class I human carcinogen [1,2,3]. According to the International Agency for Research on Cancer, Cd can infiltrate the human body via the food chain and accumulate within the body after binding to metallothionein. This accumulation may result in acute or chronic toxicity to various organs and systems (including the liver, kidneys, lungs, testicles, brain, bones, nervous system, and blood), with only negligible Cd excreted via the kidneys [4,5]. Research indicates that chronic Cd exposure increases the risk of musculoskeletal disorders, peripheral neuropathy, altered equilibrium, poor performance with regard to visuomotor tasks, and malignancies in humans [6,7,8,9,10]. Therefore, abating Cd toxicity and decreasing Cd accumulation are vital for human and animal safety.

The toxic effects and long-term accumulation of Cd primarily affect the intestinal tract [11,12]. Cd ingestion over an extended duration worsens colitis and damages the intestinal barrier in rodents [13]. Elevated concentrations of tumor necrosis factor-α (TNF-α), interleukin-1β (IL-1β), interferon-γ (IFN-γ), and IL-17 have been observed in the intestinal tract in conjunction with alterations in intestinal morphology [14].

Selenium (Se), an essential trace element and component of selenocysteine, participates in numerous biological processes; therefore, Se supplementation may have health benefits. The results of numerous studies have shown that Se has numerous functions, encompassing antioxidative properties, cancer-resistance mechanisms, immune regulation, hypoglycemia regulation, and modulation of the intestinal microbiome [15,16,17,18]. In particular, Se supplementation positively contributes to human health by enhancing the immune system and preventing or treating cancer, cardiovascular disease, HIV infection, and inflammatory bowel disease (IBD) [19,20,21,22,23,24,25].

Se can bolster antioxidant defense mechanisms and mitigate the production of reactive oxygen species that form following Cd exposure. Additionally, Se protects cells against the detrimental effects of excessive H_2_O_2_, detoxifies heavy metals, and inhibits the oxidative modification of lipids or fats [26]. The ingestion of Se during Cd poisoning can potentially prevent Cd accumulation in cells and tissues, increase the synthesis of antioxidant selenoproteins, and inhibit Cd toxicity through the nuclear factor-like 2 (Nrf2) pathway [27]. Furthermore, Se serves as an important regulator of the immune system by inducing the production of IgG and IgM antibodies, which subsequently boost the activities of T cells and macrophages [26].

Se is predominantly found in the environment in two forms: inorganic and organic selenium [28]. Nano-Se is a new form of Se. Compared with organic Se, nano-Se has lower toxicity. These SeNPs effectively safeguard intestinal epithelial cells in both animals and humans [29,30]. Due to its notable antibacterial and cancer-resistant attributes and high bioavailability, nano-Se has been extensively applied to treat inflammation and oxidation-mediated disorders, including diabetes and cancer [31,32,33]. Moreover, research has indicated that nano-Se supplementation can effectively mitigate the cardiotoxicity of Cd exposure in rodents with myocarditis that were exposed to Cd [34]. However, comparative studies of the ameliorative effects of nano-Se on Cd-induced enterotoxicity are limited.

In this study, we aimed to assess the protective effects of nano-Se against Cd toxicity and examine its potential to alleviate Cd-aggravated colitis. The aim of this study was to provide theoretical validation for the health implications of nano-Se and to present novel perspectives for developing secure and efficient Cd detoxification techniques.

## 2. Materials and Methods

### 2.1. Animal Husbandry and Colitis Induction

**Figure 1:**
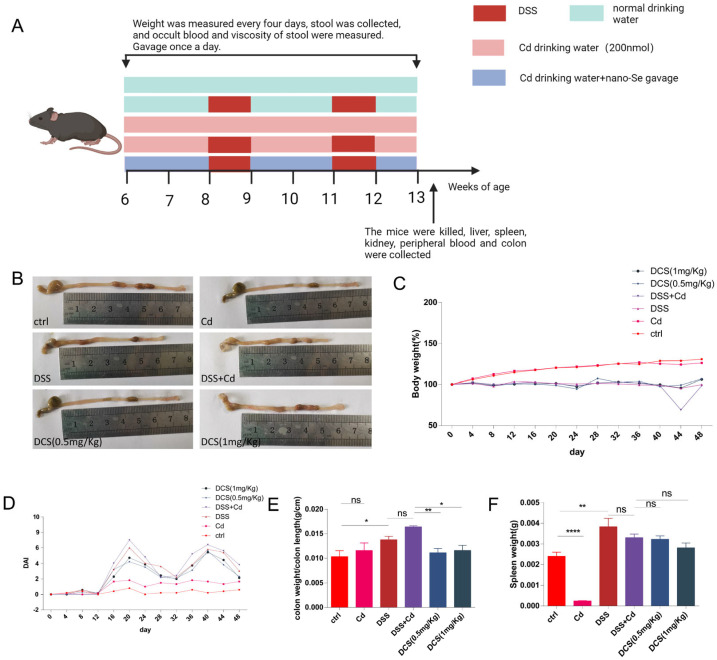
Effect of DSS-induced chronic colitis in mice. (**A**) The modeling scheme of DSS-induced chronic colitis with nano-selenium: Mice were randomly divided into control group (Ctrl), DSS group (DSS), DSS + Cd, DSS + Cd+ nano-selenium (0.5 mg/kg) group (DCS (0.5 mg/kg)), DSS + Cd+ nano-selenium (1 mg/kg) group (DCS (1 mg/kg)). Mice in the control group only received normal drinking water, while mice in the treatment groups received DSS (2% *w*/*v*) dissolved in drinking water. For nano-selenium (0.5 mg/Kg) and nano-selenium (1 mg/kg) groups, nano-selenium was administered by gavage after DSS exposure. Each cycle includes 1 week of DSS administration followed by 2 weeks of recovery with normal drinking water. The relative body weight curve (**C**) and disease activity index (DAI) score (**D**) of mice were recorded. After the modeling (end of week 7), mice were sacrificed to collect tissue and measure the length of the colon (**B**), the weight/length ratio of the colon (**E**), and spleen weight (**F**) of mice were calculated. The data are presented as means ± SEM. ns ≥ 0.05, * *p* < 0.05, ** *p* < 0.01, **** *p* < 0.0001.

The Animal Ethics Committee of Xi’an Medical University has granted approval for all the animal studies conducted in this work (No. XYLS2024104). Male C57BL/6 J mice, aged six weeks, were obtained from Hunan SJA Laboratory Animal Co., Ltd. They were kept in a controlled environment with a temperature of 23 ± 3 °C, humidity of 35 ± 5%, and a 12 h dark/light cycle in a particular pathogen-free animal facility. The mice were provided with a conventional laboratory mouse meal that had been exposed to 60Co radiation. The diet structure was obtained from SJA Laboratory Animal Co., Ltd. in Changsha, China. The mice were divided into five groups at random: (1) control treatment group (ctrl group, *n* = 5); (2) Cd exposure group (Cd group, *n* = 10); (3) dextran sulfate sodium (DSS) treatment group (DSS group, *n* = 10); (4) Cd exposure with DSS treatment group (DSS + Cd group, *n* = 10); and (5) DSS, Cd, and nano-Se (DCS group) group, with dose of 0.5 mg·kg^–1^·2 day^–1^ or 1 mg·kg^−1^·2 day^−1^ [35]. The control mice were given plain drinking water, while the DSS mice were given DSS dissolved in drinking water at a concentration of 2% *w*/*v* (Sigma-Aldrich Inc., St. Louis, MO, USA). The DSS + Cd and Cd mice were given CdCl_2_ dissolved in drinking water at a concentration of 200 nM. The DCS group was administered DSS and CdCl2 dissolved in drinking water, as well as nano-selenium by intragastric administration. In order to cause long-term inflammation of the colon, the mice were treated with Cd and nano-selenium for 2 weeks, followed by two cycles of DSS treatment (36–50 kDa; Mp Biomedicals, Santa Ana, CA, USA). Each cycle included of one week of DSS administration, followed by two weeks of normal/Cd water recovery, and ongoing nano selenium therapy. Figure 1A displays a diagram illustrating the technique. This study aimed to assess the impact of a prolonged intake of nano-Se on the intestines, specifically comparing the effects between the control and Cd groups. Additionally, the study examined how Cd worsens chronic colitis generated by DSS. Notably, our findings also revealed the influence of DSS on Cd-induced intestinal damage. The study utilized a fecal occult blood test kit (Yeasen, Shanghai, China) to evaluate the presence of fecal occult blood. Based on Jiang et al., when colitis is more severe, gross blood can be seen by the naked eye without distinguishing features, and the fecal consistency presents as watery stool [14]. To establish a disease activity index (DAI) score (Table 1) [36], we assessed the consistency of feces and the presence of hidden blood in feces every 4 days, and we measured body weight every 2 days. After the end of the second cycle, the status of the mice was observed for a week, the mice were killed, and the organs of mice were collected and weighed.

### 2.2. Histological Scoring and Periodic Acid-Schiff Staining

Colon tissues were fixed in 4% paraformaldehyde and stained with hematoxylin and eosin (H&E). Histological examinations were performed blinded using the scoring system indicated in Table 2. When colon tissue damage was severe and only the epithelium was intact, the inflammation was considered to be severe, and the lesion degree was considered to be transmural [37]. Colon sections were stained using standard PAS protocols and images were taken using a light microscope (Leica, Shanghai, China). The number of positive cells was determined under the light microscope. We also recorded the number of GCs present among epithelial cells. This analysis was also conducted blinded.

### 2.3. Analysis of Intestinal Permeability

The mice were orally administered FITC-dextran (molecular weight: 4 kDa, Sigma-Aldrich) at a dose of 500 mg/kg body weight in order to measure intestinal permeability. After administering the substance orally, blood samples were taken 4 h later. The levels of FITC-dextran were measured using a sophisticated microplate reader capable of performing many functions [38,39].

### 2.4. Quantitative Real-Time PCR

Colon tissues were subjected to RNA extraction using a Solarbio Total RNA Extraction Kit (Invitrogen, Carlsbad, CA, USA). Subsequently, cDNA synthesis was performed using a Biosharp Reverse Transcription Kit (Lanjieke Technology Co., Ltd., Hefei, China). The qRT-PCR analysis was conducted using Biosharp SYBR Green qPCR Mix (Lanjieke Technology Co., Ltd.) and a two-step procedure. The primers used for this analysis are specified in Table 3. The relative mRNA-expression levels were calculated using the 2^−ΔΔCt^ technique.

### 2.5. Serum ELISA

The blood from the orbit was obtained and subjected to centrifugation at a speed of 3000 rpm for a duration of 10 min following a clotting period of 4 h at a temperature of 4 °C. The serum was isolated and preserved for later examination of the levels of inflammatory cytokines.

### 2.6. Short-Chain Fatty Acid (SCFA) Analysis

Using a gas chromatography system called 2010 Plus, fitted with a DB-FFAP column (30 m × 0.25 mm, 5 μm), SCFAs were evaluated by following a method that had been published before, with some small changes [40]. The temperature of the gasification chamber, detector, intake, and column were all 240 °C. Before injection, each sample (1 μL) underwent eight solvent washes, followed by an additional 10 washes following injection. This process was performed three times for each sample. The SCFA concentrations were determined using conventional calibration curves.

### 2.7. Gut Microbiota Analysis

Fecal samples were dispatched to Personal Biotechnology Co., Ltd. (Shanghai, China) for the purpose of 16S rDNA gene sequencing. The XX platform (Illumina, San Diego, CA, USA) was used to undertake paired-end sequencing of DNA fragments from the microbial population. The microbiome was analyzed using Paiseno Genes Cloud Platform (www.genescloud.cn) for bioinformatics analysis. The sequencing primers utilized were as follows: the forward primer sequence was 5′-ACTCCTACGGGAGGCAGCA-3′ while the reverse primer sequence was 5′-ACTACTACHVGGGTWTCTAAT-3′. We performed sequencing specifically targeting the 16S_V3V4 region, using the gg_13 database and employing the DADA2 method as the main approach. The credibility of the experimental data was evaluated by employing rarefaction and species–accumulation curves.

### 2.8. Statistical Analysis

The data were analyzed using GraphPad Prism version 7 (GraphPad Software, Inc., La Jolla, CA, USA) and were reported as the mean ± SEM. The statistical significance of the differences was assessed using a one-way or two-way analysis of variance (ANOVA) for multiple groups and the Student’s *t*-test for two groups. A significance level of *p* < 0.05 was used.

## 3. Results

### 3.1. Nano-Se Relieves the Chronic Colitis Induced by DSS in Mice

Colitis was confirmed in the mice in the DSS and DSS + Cd groups based on weight loss and DAI score (Figure 1C,D). The DCS group exhibited significantly reduced colonic inflammation compared to the DSS and DSS + Cd groups, indicating that nano-Se supplementation effectively alleviated chronic colitis induced by combined treatment with DSS and Cd. Additionally, the DSS + Cd group showed significantly shorter colon lengths compared to the DCS group, as shown in Figure 1B.

### 3.2. Nano-Se Alleviates Colonic Inflammatory Cytokines Expression

**Figure 2:**
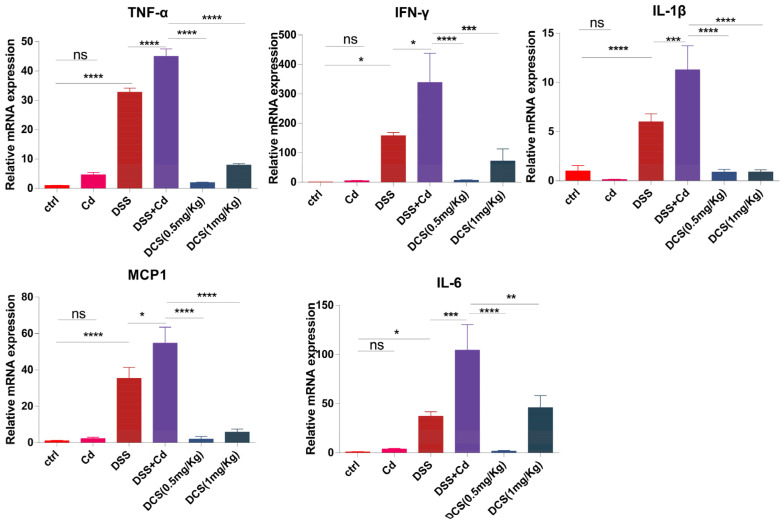
Nano-selenium treatment reduced colonic inflammatory cytokines expression. Relative mRNA levels of IL-1β, IL-6, MCP1, TNF-α, and IFN-γ in the colon were measured through qPCR (*n* = 8–12). The data are presented as means ± SEM. ns ≥ 0.05, * *p* < 0.05, ** *p* < 0.01, *** *p* < 0.001, **** *p* < 0.0001.

We assessed the levels of IL-1β, IL-6, TNF-α, monocyte chemotactic protein-1 (MCP1), and IFN-γ in the colon to measure the inflammatory reaction following nano-Se therapy. Consistent with expectations, DSS stimulated the mRNA expression of IL-1β, IL-6, IL-17, TNF-α, and IFN-γ, as shown in Figure 2. In addition, when compared to the treatment of DSS + Cd, nano-Se had a considerable inhibitory effect on the expression of inflammatory cytokines in the colon, as shown in Figure 2.

### 3.3. Nano-Se Protects the Intestinal Barrier

**Figure 3:**
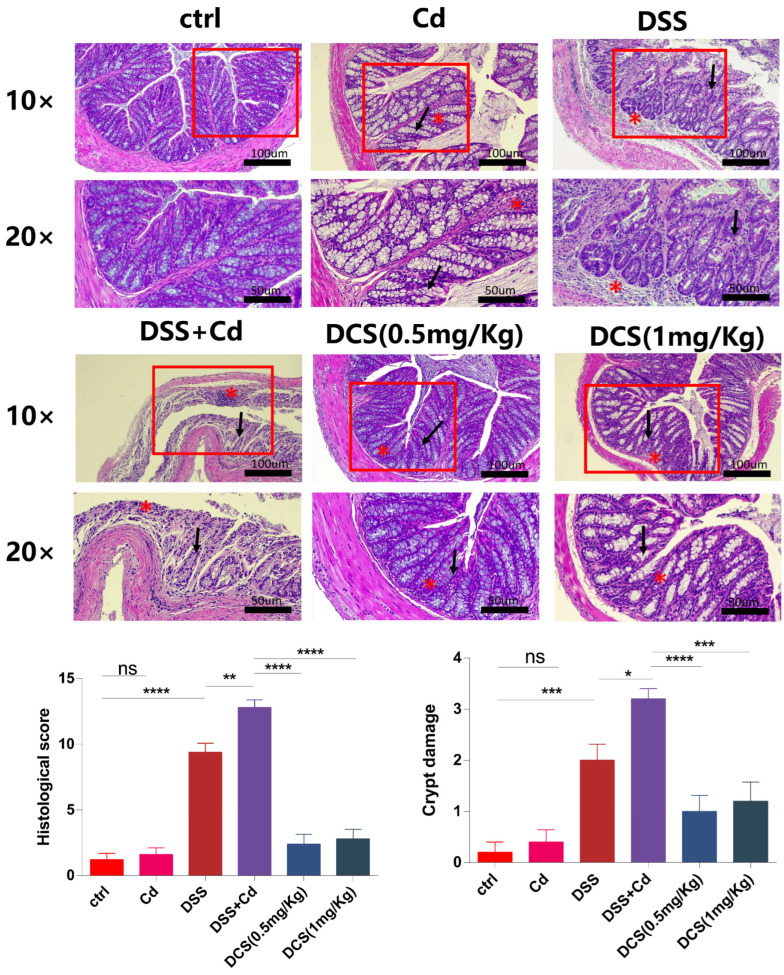
Nano-selenium treatment protects the intestinal barrier in mice. Microscopic appearance by using H&E staining of the colon tissues. The inflammatory cell infiltration is indicated with (asterisk). The crypt damage is indicated with (arrow). The data are presented as means ± SEM. ns ≥ 0.05, * *p* < 0.05, ** *p* < 0.01, *** *p* < 0.001, **** *p* < 0.0001.

**Figure 4:**
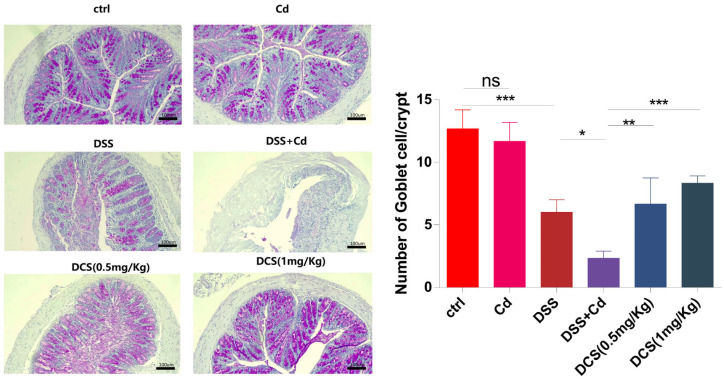
The PAS stain of goblet cells in the colon. Quantification of goblet cell depletion in the colon (*n* = 6). The data are presented as means ± SEM. ns ≥ 0.05, * *p* < 0.05, ** *p* < 0.01, *** *p* < 0.001.

**Figure 5:**
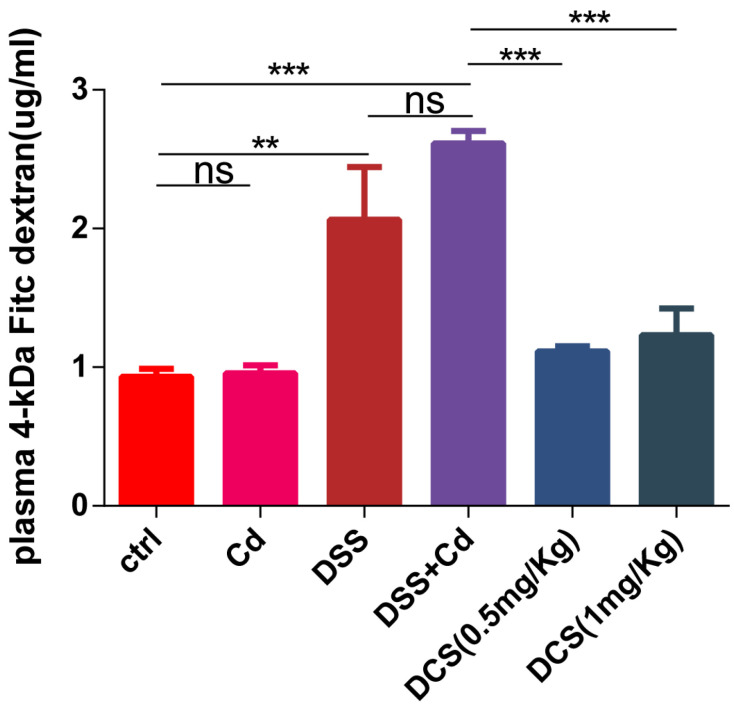
Serum FITC content. The data are presented as means ± SEM (*n* = 3). The data are presented as means ± SEM. ns ≥ 0.05, ** *p* < 0.01, *** *p* < 0.001.

Histological analysis of the colon revealed typical signs of inflammation following DSS treatment, as characterized by massive inflammatory cell infiltration and crypt damage (Figure 3). The DCS group showed significantly lower microscopic histological scores than the DSS + Cd group. Notably, only the DSS + Cd group showed more serious crypt damage in the colon. H&E staining revealed crypt damage in the DSS + Cd group. PAS staining showed that the number of GCs was markedly lower in the DSS + Cd group than in the other groups (Figure 4). In addition, the detection of intestinal leakage with FITC showed that the serum FITC contents in the control and FITC groups were significantly lower than those in the DSS and DSS + Cd groups (Figure 5).

### 3.4. Nano-Selenium Relieves Colitis-Related Extraintestinal Inflammation

**Figure 6:**
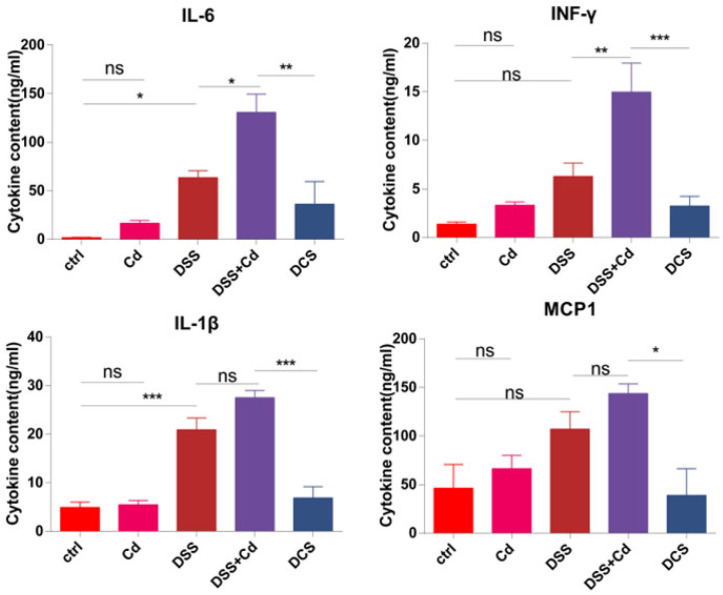
Effect of nano-Se on plasma inflammatory cytokines. The relative levels of IL-1β, IL-6, MCP1, and IFN-γ in plasma were measured by using ELISA. The data are presented as means ± SEM (*n* = 3). The data are presented as means ± SEM. ns ≥ 0.05, * *p* < 0.05, ** *p* < 0.01, *** *p* < 0.001.

In order to assess the impact of nano-Se on extraintestinal inflammation, we quantified the levels of inflammatory cytokines in the serum following various treatments. The levels of proinflammatory cytokines, including as IL-1β, IL-6, MCP1, TNF-α, and IFN-γ, were considerably increased when DSS and Cd were administered together. However, treatment with nano-Se resulted in a decrease in the levels of these cytokines (Figure 6). The results indicate that the use of nano-Se reduced inflammation outside the intestines.

### 3.5. Effect of Nano-Selenium on SCFA Production

**Figure 7:**
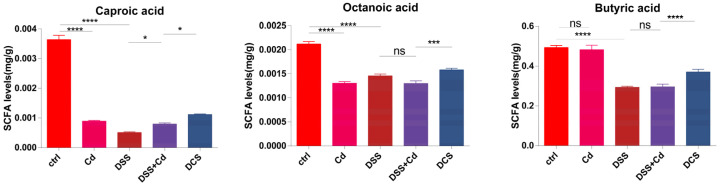
Nano-Se affects the content of SCFAs in feces. The data are presented as means ± SEM. ns ≥ 0.05, * *p* < 0.05, *** *p* < 0.001, **** *p* < 0.0001.

SCFAs are produced by the gut microbiota, and increasing SCFA contents promotes good health and protects colon tissue from DSS-induced damage [41]. To further explore the effect of nano-Se on the intestinal barrier, we measured the SCFA contents in mouse feces. The fecal concentrations of caproic acid, butyric acid, and octanoic acid in the DCS group were significantly higher than those in the DSS + Cd group (Figure 7).

### 3.6. Nano-Se Improves DSS-Induced Changes in the Structure of Gut Microbiota

**Figure 8:**
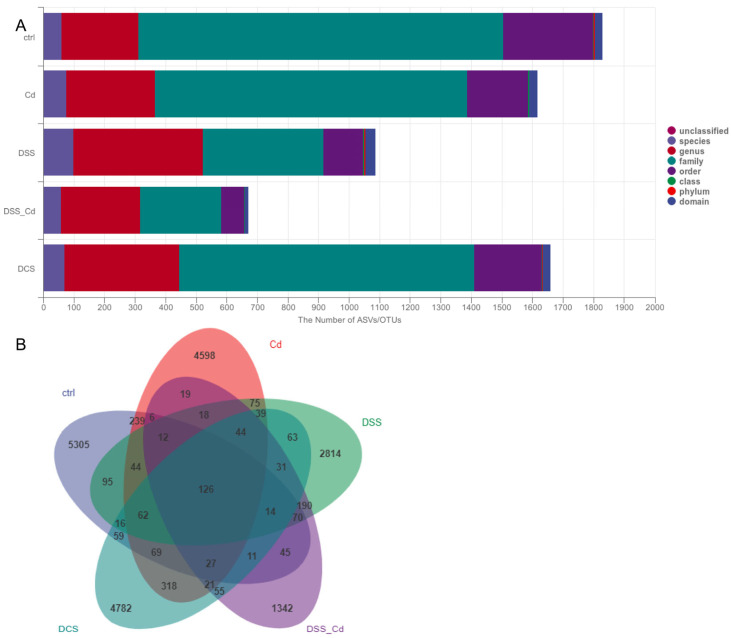
Notes on species taxonomy and analysis of ASV Venn diagram. (**A**) Notes on species taxonomy; (**B**) ASV Venn diagram.

The rarefaction and species–accumulation curves of the fecal samples exhibited a predilection for an initial increase in species diversity, followed by a subsequent plateau. The experiment yielded 47,749 nucleotides with an average read length of 0.02947, indicating that the acquired data are reliable (Figure S1A–C). The findings from the ASV Venn plot and alpha-diversity analysis indicate that the species diversity and community richness of the gut microbiota were significantly lower in the mice with colitis in the DSS + Cd group than in the other groups (Figure 8A,B and Figure S2). Conversely, administering nano-Se via gastric irrigation to mice that were fed a high-fat diet resulted in a substantial increase in both the diversity and community richness of the gut microbiota (Figure S2). Principal component analysis (PCA) and non-metric multidimensional scaling (NMDS) using the Bray–Curtis distance algorithm indicate that the mice in the DCS group were considerably separated from those in the control, DSS, and DSS + CD groups. Specifically, PC1 and PC2 accounted for 85.5% of the overall variation and 72.6% and 12.9% of the variation, respectively (Figure S3A,B). Furthermore, we examined distinctions among the groups using the analysis of similarities method and Bray–Curtis distance algorithm, finding that the DCS group was not significantly different from the control, DSS, and DSS + CD groups (Figure S3C,D). These findings are consistent with the results of our NMDS analysis. Hence, the mice groupings utilized in this experiment were deemed to be ideal, and the microbiota investigations were conducted based on these classifications. The colitis mice that were exposed to nano-selenium were accurately categorized, and a microbiota study was conducted based on these categorizations.

**Figure 9:**
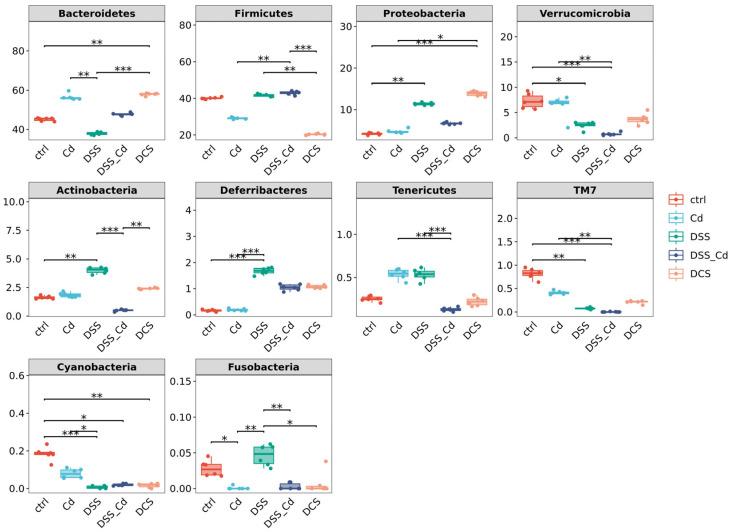
Analysis of species difference at the phylum level of nano-selenium intervention in DSS-induced enteritis. The data are presented as means ± SEM. * *p* < 0.05, ** *p* < 0.01, *** *p* < 0.001.

**Figure 10:**
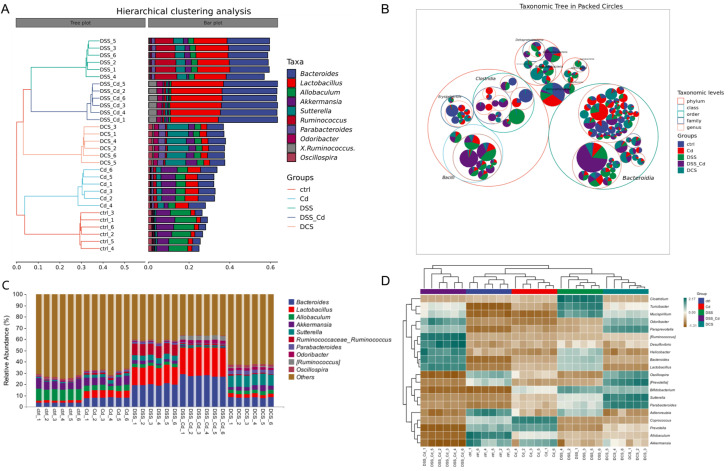
Analysis of dominant species based on relative abundance family level. (**A**) relative abundance information of species at genus level; (**B**) classification-level tree diagram; (**C**) hierarchical cluster analysis; (**D**) species composition heatmap.

**Figure 11:**
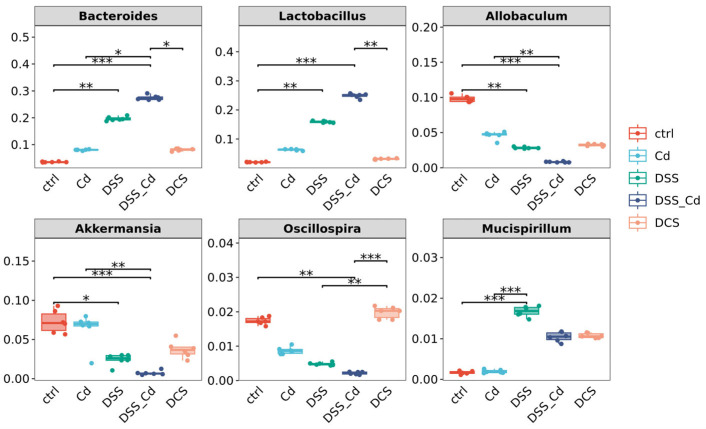
Analysis of species difference at the genus level of nano-selenium in intervention of DSS-induced enteritis. The data are presented as means ± SEM. * *p* < 0.05, ** *p* < 0.01, *** *p* < 0.001.

Our analyses included taxonomic composition, species composition heatmap, hierarchical clustering, and phylogenetic analysis. The analyses of Krona species composition indicated variation at the phylum level (Figure 9 and Figure S4A–D). *Firmicutes* and *Bacteroides* are closely associated with enteritis. Che-SeNP treatment significantly increased the relative abundances of *Bacteroides* and decreased the relative abundance of *Firmicutes*. At the genus level (Figure 10A–D and Figure 11), the relative abundances of *Bacteroides* and *Lactobacillus* in the DCS group were lower than those in the DSS + Cd group, whereas the relative abundances of *Allobaculum, Akkermansia*, and *Oscillospira* in the DCS group were higher than those in the DSS + Cd group.

### 3.7. Conducting a Correlation Analysis to Examine the Relationship between Specific Species and Indications of Colitis

**Figure 12:**
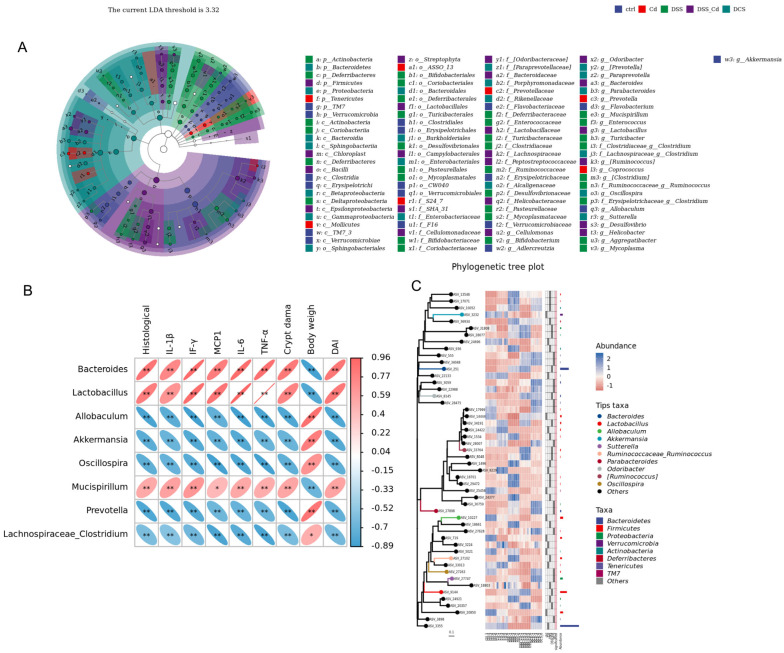
Correlation analysis between the relative abundance of species and related indexes of enteritis. (**A**) LefSe analysis; (**B**) genus-level evolutionary tree; (**C**) correlation analysis between the relative abundance of species and enteritis-related indicators. The data are presented as means ± SEM. * *p* < 0.05, ** *p* < 0.01.

The correlations between the relative abundances of the different species and the markers of colitis were studied at the gene level (Figure 12A–C). The results showed that changes in the abundances of *Bacteroides* and *Lactobacillus* correlated positively with the expression of proinflammatory factors. Changes in *Allobaculum, Akkermansia*, and *Oscillospira* abundances correlated negatively with the expression of proinflammatory factors (Figure 12B). By downregulating the relative abundance of *Bacteroides* and *Lactobacillus* and upregulating the relative abundance of *Allobaculum, Akkermansia*, and *Oscillospira*, the expression levels of pro-inflammatory factors in mice with colitis were significantly inhibited, thereby improving the intestinal inflammation caused by long-term DSS treatment.

## 4. Discussion

Cd, an acknowledged environmental contaminant, can be assimilated by plants in the soil before entering the food chain, thereby posing a threat to human health [42]. Cd is associated with colitis severity, and Se supplementation can alleviate colitis by reducing the cellular and systemic harm caused by Cd [14,34]. Additionally, previous research indicated that nano-Se can mitigate the Cd-induced dysregulation of inflammatory genes [43]. However, the role of nano-selenium in antagonizing cadmium-aggravated intestinal injury is unclear. Thus, the objectives of this study were to determine whether nano-Se can alleviate Cd-aggravated colitis and to elucidate the mechanism whereby Se relieves colitis.

Chronic Cd exposure induced intestinal injury and inflammation in a previous murine model of colitis [14]. The ability of Se to counteract the deleterious effects of Cd was supported by prior data [44]. Supplemental Se prevents Cd-induced oxidative stress in the liver, reduces Cd-induced renal toxicity and DAI scores, increases colon length, and alleviates colitis symptoms in rodents [45]. In this study, nano-Se supplementation resulted in significantly lower DAI scores and weight loss than found in the DSS + Cd group, suggesting that the incidence of colitis was stably reduced.

Colitis frequently induces numerous pro-inflammatory factors and disrupts intestinal tissue integrity [46]. Thus, we evaluated the gene-expression levels of IL-6, IL-1β, TNF-α, IFN-γ, and MCP1, finding that their expression levels increased in response to Cd exposure but were substantially downregulated by nano-Se intervention. Serum analysis confirmed that Cd induced inflammation and that nano-Se supplementation alleviated this inflammation. Our results indicate that nano-Se effectively mitigated Cd-induced intestinal and systemic inflammation.

In addition to studying inflammation, we investigated the effects of Cd on the intestinal barrier. An intact intestinal mucosa prevents the infiltration of exogenous toxins and dietary contaminants into the portal venous circulation by acting as a barrier [47]. The intestinal barrier is maintained at the cellular level through tight junctions, complexes of transmembrane proteins (e.g., occludin and claudin), and scaffold proteins (e.g., ZO-1) [48]. Additionally, the adhesion-related proteins E-cadherin and β-catenin are crucial for maintaining the intestinal barrier [49]. An extended exposure to Cd in potable water decreased the number of GCs, disrupted the structural integrity of the intestines, and inhibited the expression and subcellular localization of adhesion-related and tight junction proteins. These effects were reversed in mice supplemented with nano-Se, suggesting that intestinal barrier function (facilitating the passage of substances) was compromised by long-term Cd exposure but protected by nano-Se supplementation. In this study, oral Cd administration increased serum FITC concentrations, whereas Se supplementation decreased serum FITC levels. Inflammation was induced by intestinal barrier disruption, which facilitated the entry of exogenous inflammatory mediators such as DSS. Our hypothesis posits that while maintaining the integrity of the intestinal barrier, nano-Se might mitigate inflammation in the intestines and throughout the body.

Cd exposure can disrupt the dynamic equilibrium of the gastrointestinal microbiota in mice, resulting in an increased prevalence of Bacteroidetes relative to Firmicutes and a suppressed growth of probiotics, including that of *Bifidobacteria* and *Lactobacillus* [50,51]. Substantial evidence suggests that the microbiome contributes to host diseases and is strongly correlated with the onset of IBD (35). Therefore, one potential mechanism whereby nano-Se ameliorates Cd-aggravated colitis involves the gut microbiota, so we examined the effects of nano-Se on the gut microbiota in Cd-treated rodents. Using nano-Se to treat colitis in mice substantially augmented the alpha diversity within the gut microbiota, as determined by calculations based on the Chao index, in contrast to our findings with the DSS + Cd group. The phylum-level microbial analysis revealed that rodents subjected to nano-Se treatment exhibited a notable increase in Firmicutes and a decrease in the relative abundance of Bacteroidetes compared with the corresponding abundances in the DSS group. When studying the DSS group at the genus level, we found that nano-Se significantly increased the relative abundances of *Allobaculum, Akkermansia*, and *Oscillospira*. Colitis frequently develops in response to interactions between microorganisms and SCFAs. Consequently, the intestinal microbiota is likely one of the primary targets of nano-Se during the treatment of colitis.

SCFAs are byproducts of carbohydrate metabolism by the intestinal microbiota. SCFAs are obtained from dietary fibers, including resistant starch, inulin, and polysaccharides [52]. SCFAs are predominantly absorbed by the intestinal epithelial cells in the colon and serve as essential energy sources for fundamental cellular processes [53]. SCFAs serve essential functions in maintaining intestinal homeostasis in numerous ways, including by increasing the expression of the tight junction proteins, ZO-1, and occludin in colonic intestinal cells and by substantially decreasing inflammatory responses [54]. SCFAs promote overall health by facilitating the formation of GCs [55]. Nano-Se substantially increased the concentrations of butyric, acetic, and propionic acid in the feces of mice with DSS-induced colitis. Consequently, SCFAs constitute a focal point in the mechanism through which nano-Se operates.

## 5. Conclusions

Cd can destroy the integrity of the intestinal epithelial barrier and intestinal homeostasis, reduce the content of probiotics, and increase intestinal inflammation. Nano-Se can maintain the integrity of the intestinal epithelial barrier and ameliorate Cd damage to intestinal health by regulating short-chain fatty acids and microorganisms in the intestine. In this study, nano-Se was used at a laboratory dose; the suitability of this dose in humans requires further research. This study cannot be applied to the treatment human patients with colitis. 

## Figures and Tables

**Table 1:** DAI scoring system.

Score	Body Weight Loss	Fecal Consistency	Fecal Occult Blood
0	0	Negative	Negative
1	1–5%	Soft stool	Light blue
2	5–10%	Mucoid stool	Blue
3	10–20%	Watery stool	Dark blue
4	>20%		Gross blood

**Table 2:** Histological scoring system.

Score	Severity of Inflammation	Depth of Injury	Crypt Damage	Percentage of the Involved Area
0	None	None	None	
1	Slight	Mucosal	Basal 1/3 damaged	1–10%
2	Moderate	Mucosal and submucosal	Basal 2/3 damaged	10–25%
3	Severe	Transmural	Only surface epithelium intact	25–50%
4			Entire crypt and epithelium lost	50–100%

**Table 3:** Primers for real-time PCR analysis.

Gene	Sense	Anti-Sense
IL-1β	GCAACTGTTCCTGAACTCAACT	ATCTTTTGGGGTCCGTCAACT
TNF-α	GCCTCCTCACCCACCACCATCA	CCAAGTAGACCTGCCCAGACT
IL-6	GAGGATACCACTCCCAACAGACC	AAGTGCATCATCGTTGTTCATACA
MCP1	TTAAAAACCTGGATCGGAACCAA	GCATTAGCTTCAGATTTACGGGT
IFN-γ	ATGAACGCTACACACTGCATC	CCATCCTTTTGCCAGTTCCTC
β-actin	GGCTGTATTCCCCTCCATCG	CCAGTTGGTAACAATGCCATGT

## Data Availability

The original contributions presented in the study are included in the article/Supplementary Material, further inquiries can be directed to the corresponding author/s.

## Supplementary Materials

The following supporting information can be downloaded at: https://www.mdpi.com/article/10.3390/nu16091330/s1, Figure S1: species accumulation curve, abundance grade curve, sequence length distribution, and rarefaction curves analysis; Figure S2: Alpha diversity index analysis; Figure S3: PCA analysis and Beta diversity analysis; Figure S4: Analysis of dominant species based on relative abundance phylum level.
