# Supplementary material for: Nano-Selenium Alleviates Cd-Induced Chronic Colitis through Intestinal Flora
Source: Nutrients. 2024 Apr 28;16(9):1330. doi: 10.3390/nu16091330 (PMC11085897; doi:10.3390/nu16091330)
Supplement: Supplementary file 1 [file nutrients-16-01330-s001.zip › nutrients-2949103-supplementary.pdf]

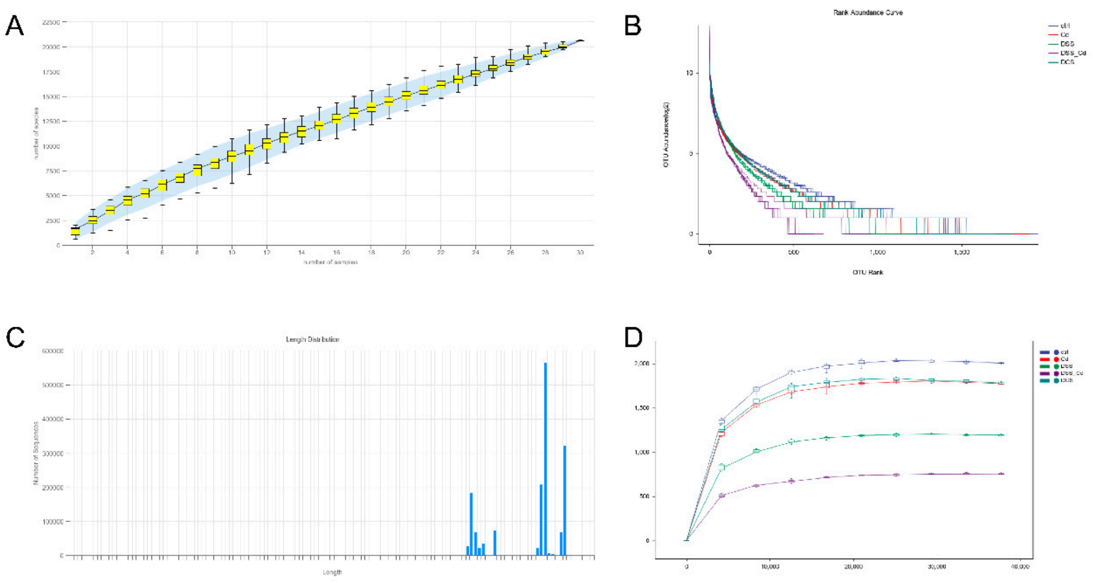

Figure S1 species accumulation curve, abundance grade curve, sequence length distribution, and

rarefaction curves analysis. A, species accumulation curve; B, abundance grade curve; C, sequence length distribution; D, rarefaction curves

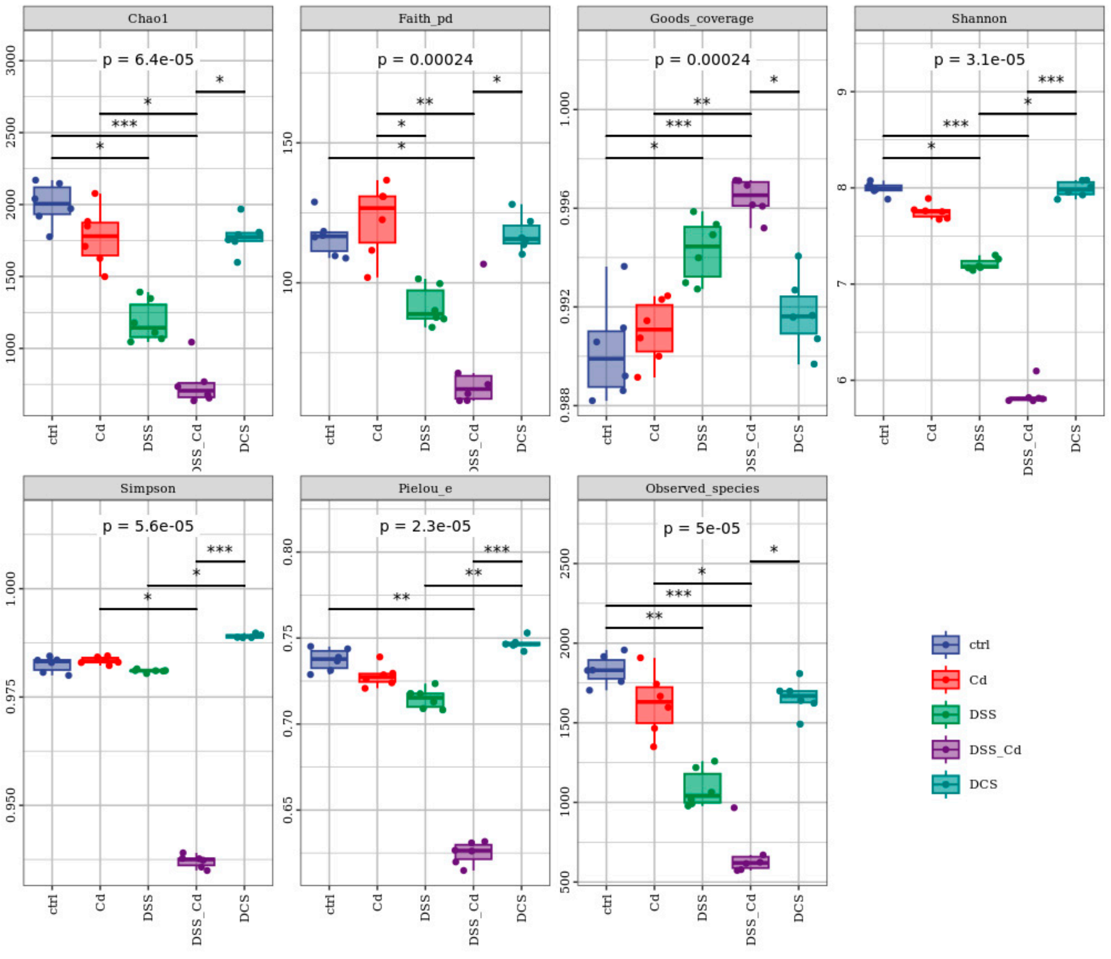

Figure S2 Alpha diversity index analysis

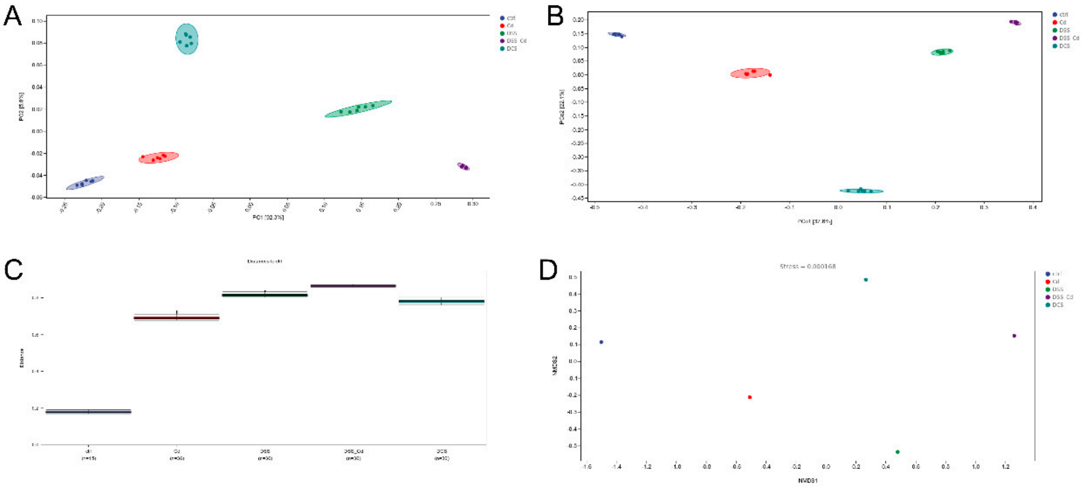

Figure S3 PCA analysis and Beta diversity analysis. A, PCA analysis; B, PCoA analysis; C, NMDS analysis; D, Analysis of differences between groups

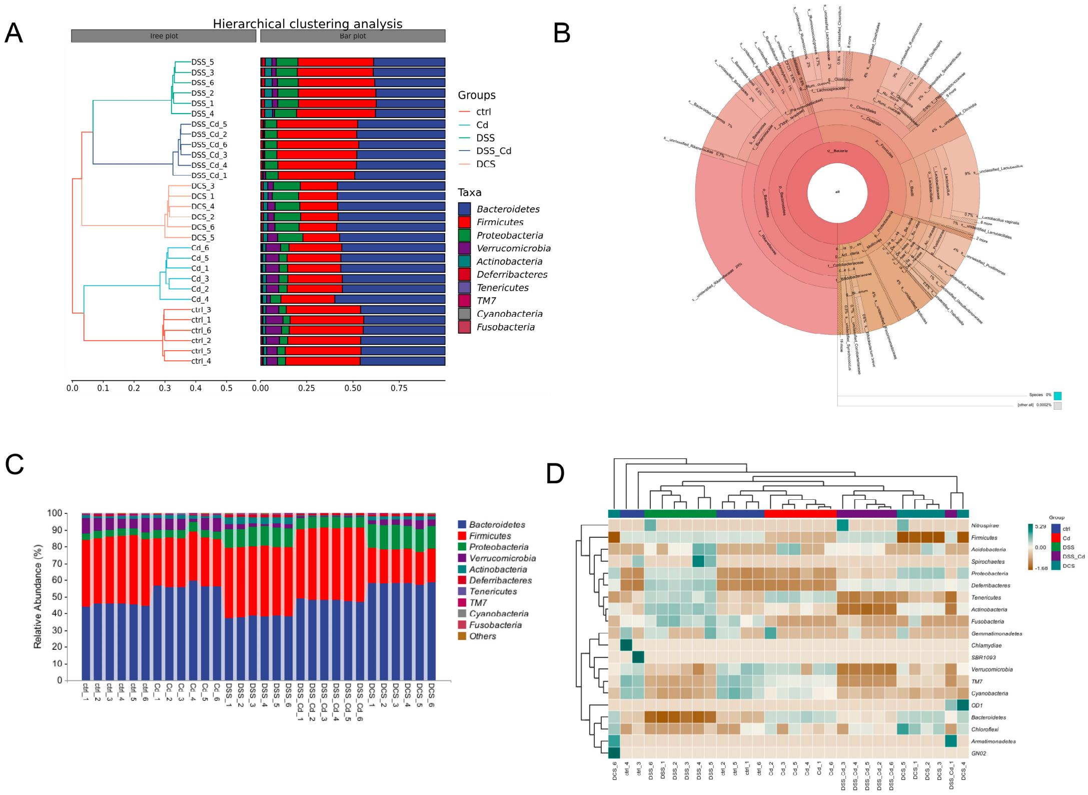

Figure S4: Analysis of dominant species based on relative abundance phylum level. A, hierarchical cluster analysis; B, Krona species composition analysis; C, relative abundance information of species at phylum level; D, species composition heat map
